# Supplementary material for: The Protective Effect against Lung Injury of Phytosome Containing the Extract of Purple Waxy Corn Tassel in an Animal Model of PM2.5-Induced Lung Inflammation
Source: Foods. 2024 Oct 13;13(20):3258. doi: 10.3390/foods13203258 (PMC11507556; doi:10.3390/foods13203258)
Supplement: Supplementary file 1 [file foods-13-03258-s001.zip › foods-3251020-supplementary.pdf]

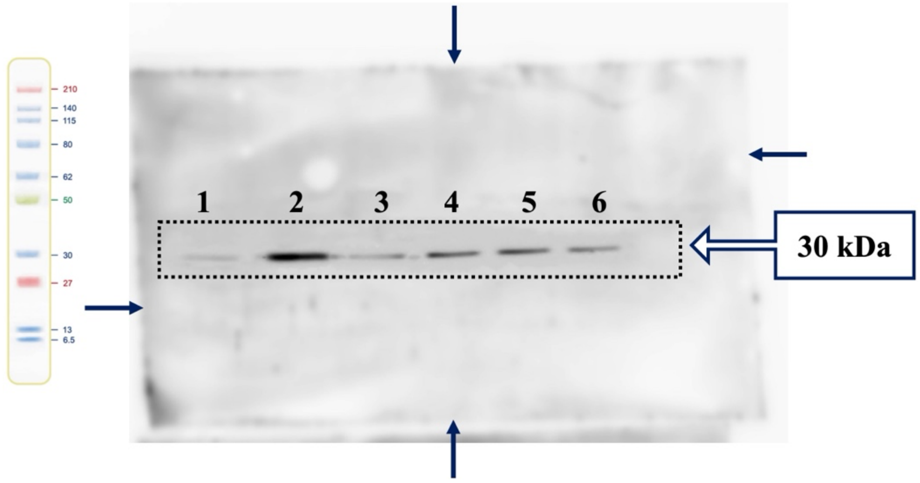

Figure S1: Western blotting was used to detect the expression of IL-1β (30 kDa) in lung tissue. The groups included: (1) Naïve control, (2) Respiratory inflammation + vehicle, (3) Respiratory inflammation + prednisolone 1 mg/kg BW, (4) Respiratory inflammation + PPT 100 mg/kg BW, (5) Respiratory inflammation + PPT 200 mg/kg BW, and (6) Respiratory inflammation + PPT 400 mg/kg BW. The full-length membranes are shown, with membrane edges indicated by the dark blue arrows.

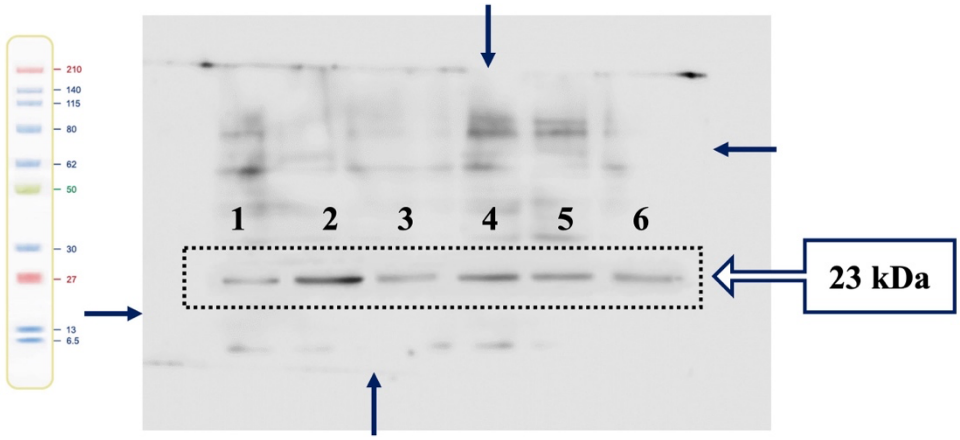

Figure S2: Western blotting was used to detect the expression of IL-6 (23 kDa) in lung tissue. The groups included: (1) Naïve control, (2) Respiratory inflammation + vehicle, (3) Respiratory inflammation + prednisolone 1 mg/kg BW, (4) Respiratory inflammation + PPT 100 mg/kg BW, (5) Respiratory inflammation + PPT 200 mg/kg BW, and (6) Respiratory inflammation + PPT 400 mg/kg BW. The full-length membranes are shown, with membrane edges indicated by the dark blue arrows.

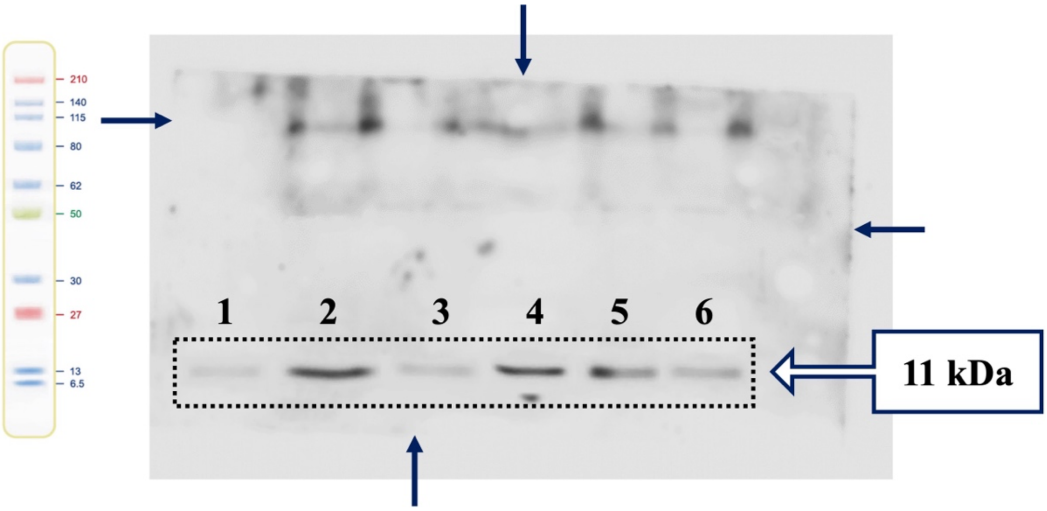

Figure S3: Western blotting was used to detect the expression of IL-8 (11 kDa) in lung tissue. The groups included: (1) Naïve control, (2) Respiratory inflammation + vehicle, (3) Respiratory inflammation + prednisolone 1 mg/kg BW, (4) Respiratory inflammation + PPT 100 mg/kg BW, (5) Respiratory inflammation + PPT 200 mg/kg BW, and (6) Respiratory inflammation + PPT 400 mg/kg BW. The full-length membranes are shown, with membrane edges indicated by the dark blue arrows.

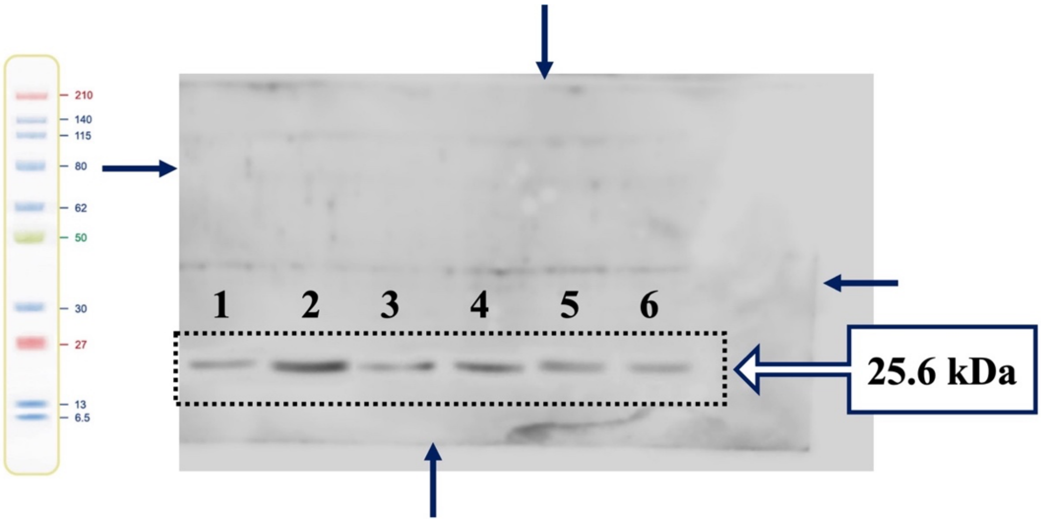

Figure S4: Western blotting was used to detect the expression of TNF-α (25.6 kDa) in lung tissue. The groups included: (1) Naïve control, (2) Respiratory inflammation + vehicle, (3) Respiratory inflammation + prednisolone 1 mg/kg BW, (4) Respiratory inflammation + PPT 100 mg/kg BW, (5) Respiratory inflammation + PPT 200 mg/kg BW, and (6) Respiratory inflammation + PPT 400 mg/kg BW. The full-length membranes are shown, with membrane edges indicated by the dark blue arrows.

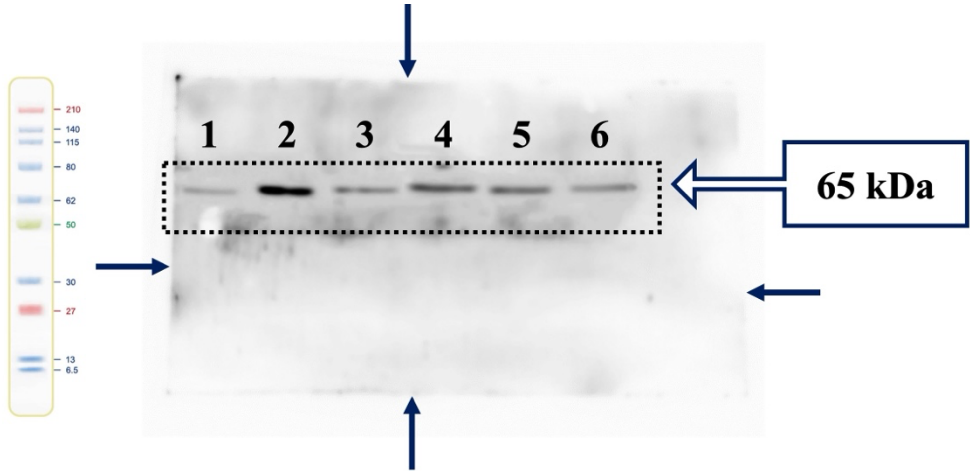

Figure S5: Western blotting was used to detect the expression of NF-κB (65 kDa) in lung tissue. The groups included: (1) Naïve control, (2) Respiratory inflammation + vehicle, (3) Respiratory inflammation + prednisolone 1 mg/kg BW, (4) Respiratory inflammation + PPT 100 mg/kg BW, (5) Respiratory inflammation + PPT 200 mg/kg BW, and (6) Respiratory inflammation + PPT 400 mg/kg BW. The full-length membranes are shown, with membrane edges indicated by the dark blue arrows.

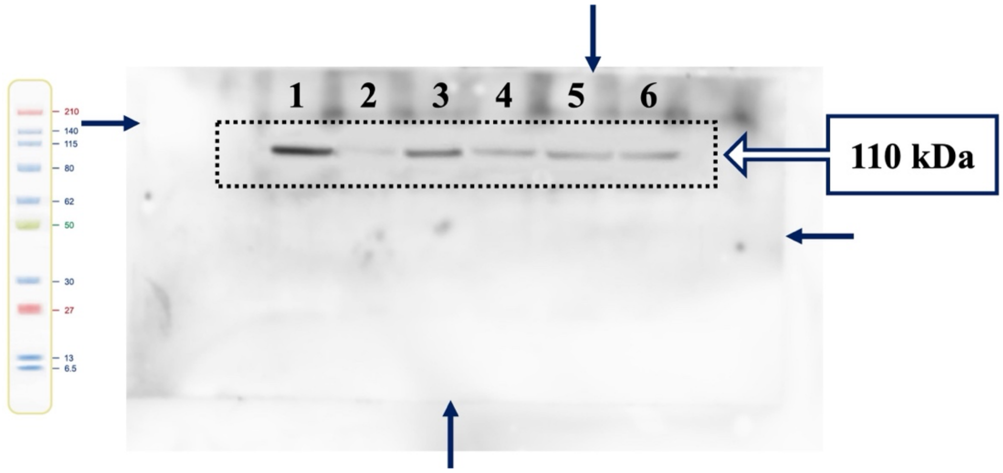

Figure S6: Western blotting was used to detect the expression of sirtuins (110 kDa) in lung tissue. The groups included: (1) Naïve control, (2) Respiratory inflammation + vehicle, (3) Respiratory inflammation + prednisolone 1 mg/kg BW, (4) Respiratory inflammation + PPT 100 mg/kg BW, (5) Respiratory inflammation + PPT 200 mg/kg BW, and (6) Respiratory inflammation + PPT 400 mg/kg BW. The full-length membranes are shown, with membrane edges indicated by the dark blue arrows.
